# High Rate and Long Lifespan Sodium-Organic Batteries Using Pseudocapacitive Porphyrin Complexes-Based Cathode

**DOI:** 10.1007/s40820-021-00593-8

**Published:** 2021-02-14

**Authors:** Xi Chen, Xin Feng, Bo Ren, Liangzhu Jiang, Hongbo Shu, Xiukang Yang, Zhi Chen, Xiujuan Sun, Enhui Liu, Ping Gao

**Affiliations:** 1 Key Laboratory of Environmentally Friendly Chemistry and Application of Ministry of Education, Key Laboratory for Green Organic Synthesis and Application of Hunan Province, College of Chemistry, Xiangtan University, Xiangtan, 411105 People’s Republic of China; 2 International Collaborative Laboratory of 2D Materials for Optoelectronics Science and Technology of Ministry of Education, Institute of Microscale Optoelectronics, College of Chemistry and Enviromental Engineering, Shenzhen University, Shenzhen, 518060 People’s Republic of China

**Keywords:** Organic cathode, Sodium-organic batteries, Porphyrin complex, Rechargeable batteries, Pseudocapacitive effect

## Abstract

**Highlights:**

- Functionalized porphyrin complexes are proposed as new pseudocapacitive cathodes for SIBs based on four-electron transfer.
- The presence of copper(II) ion partially contributes the charge storage and significantly stabilizes the structure of porphyrin complex for electrochemical energy storage.
- The electrochemical polymerization of porphyrin complex through the ethynyl groups in self-stabilization process contributes to high rate capability and excellent cycling stability.

**Abstract:**

Sodium-organic batteries utilizing natural abundance of sodium element and renewable active materials gain great attentions for grid-scale applications. However, the development is still limited by lack of suitable organic cathode materials with high electronic conductivity that can be operated stably in liquid electrolyte. Herein, we present 5,15-bis(ethynyl)-10,20-diphenylporphyrin (DEPP) and [5,15-bis(ethynyl)-10,20-diphenylporphinato]copper(II) (CuDEPP) as new cathodes for extremely stable sodium-organic batteries. The copper(II) ion partially contributes the charge storage and significantly stabilizes the structure of porphyrin complex for electrochemical energy storage. In situ electrochemical stabilization of organic cathode with a lower charging current density was identified which enables both improved high energy density and power density. An excellent long-term cycling stability up to 600 cycles and an extremely high power density of 28 kW kg^−1^ were achieved for porphyrin-based cathode. This observation would open new pathway for developing highly stable sodium-organic cathode for electrochemical energy storage.
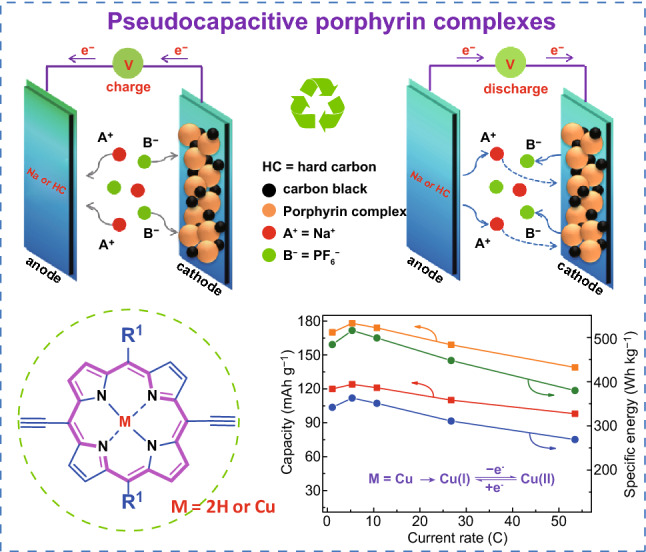

**Supplementary Information:**

The online version of this article (10.1007/s40820-021-00593-8) contains supplementary material, which is available to authorized users.

## Introduction

With the growing demand for sustainable and affordable energy storage technologies, researchers are devoted to finding an alternative to lithium-ion batteries (LIBs) for grid-scale energy storage applications [1–4]. The sodium-ion batteries (SIBs) have received great attention owing to its high performance comparable to LIBs and affordability [3, 5]. Inorganic compounds of layered sodium transition metal oxides and sodium polyanions have been extensively investigated as cathodes in SIBs, which imply that the sodium-based inorganic compounds could be potentially compared to their lithium analogues [6–8]. Highly reversible capacity was achieved in layered metal oxides but it showed poor lifespan due to the unstable structure during cycling and air sensitivity property [9–11]. The polyanions with intrinsic stable structure offer better cycling life; however, only a moderate energy density can be delivered owing to the limited sodium sites in the crystal structure and poor electronic conductivity [12, 13]. Therefore, developing suitable host materials with the combination of multiple sodium sites and stable structure is critical for high-performance SIBs [14]. In addition, the emphases on low cost, environmental benefit, and long-term cyclability are of vital importance for large scale energy storage applications.

Organic molecules are composed of elements such as C, H, N, O, S which are available in natural biomass. Unlimited sodium charge storage capabilities are feasible by using different combinations of these elements, such as conjugated molecules, covalent organic frameworks (COFs), and metal organic frameworks (MOFs) [15–19]. From a perspective of material sustainability, sodium rechargeable batteries with organic electrode materials can offer a good option regarding the future energy consumption and recyclability. In addition, high energy densities and good cycling stability are expected due to the advantages of organic compounds that are structurally diverse and can easily be functionalized through different synthetic routes [20, 21]. Moreover, organic electrode materials do not strictly depend on particular ions but are based on charge transfer reaction of redox centers of organic molecules to accommodate a large radius of sodium ions [22]. For better sodium-organic batteries, the development of stable cathode with multiple electrons transfer is of great significance and has gained widespread attention. Disodium rhodizonate was reported as a high-performance cathode for organic batteries owing to the reversible storage of four-sodium in the lattice, enabling a high theoretical capacity of 501 mAh g^−1^; however, capacity decay was remarkable upon cycling and the redox potential was relatively low as a cathode [16]. Wang et al. presented a serial conjugated polymer by tailoring the properties of organic molecules, where the strong π-π stacking in poly (pentacenetetrone sulfide) demonstrated excellent cycling stability and high power density. Nevertheless, the redox potential of sodium with carbonyl group was still too low as a cathode (< 2.0 V vs. Na^+^/Na) [23]. Eckert et al. [17] reported that porous organic electrode consisting of benzene and triazine rings showed both high energy density and long-term cycling life in sodium-organic batteries thanks to the bipolar property and the stable structure of electrode. Nevertheless, the wide application of organic electrode materials for energy storage system (EES) is still restricted by the rare organic molecules which can enable high specific energy, power density, and long-term cycling life due to the low electronic conductivity and high solubility of organic molecules in liquid electrolyte [5, 24].

Conjugated porphyrin complex contains four pyrroles and its derivatives are naturally existed such as leaves of plants, which have been proposed for wide use in many fields including photocatalytics and photovoltaics [25–27]. It has been rarely reported as active materials for rechargeable batteries so far. Recently, researchers have attempted to apply the porphyrins and its derivatives to the fabrication of rechargeable batteries including but not limited to LIBs [28–34], lithium-sulfur batteries [35–37], SIBs [38], ZIBs [39], and redox-flow batteries [40–42]. The porphyrins with smaller HOMO–LUMO (highest occupied molecular orbital–lowest unoccupied molecular orbital) gaps can significantly facilitate the absorption and release of electrons and exhibit faster redox kinetics [27, 43]. Considering the solubility of porphyrins in organic electrolytes, developing functionalized porphyrins with different organic groups and their polymers is an effective strategy for designing stable electrodes, which is a precondition for ensuring a long lifespan in sodium-organic battery. It was reported that the [5,15-bis-(ethynyl)-10,20-diphenylporphinato]copper(II) (CuDEPP) became less soluble and extremely stable through chemical functionalization of introducing an ethynyl group at the meso-position of porphyrin complex [5,10,15,20-tetraphenylporphinato]copper(II) (CuTPP) [30]. The improved stability of porphyrins in common solvents was owing to the enhanced intermolecular π-cation interaction and the self-polymerization process during the initial charge–discharge processes. To the best of our knowledge, porphyrin complexes have never been studied as cathode in sodium-organic battery. Inspired by the idea of the multifunction of ethynyl group, which can enhance the stability of structure and increase the conductivity of electrode, functionalized porphyrin molecule of 5,15-bis(ethynyl)-10,20-diphenylporphyrin (DEPP) and its complex of [5,15-bis(ethynyl)-10,20-diphenylporphinato]copper(II) (CuDEPP) are developed as new cathodes for sodium-organic batteries. With a lower molecular mass compared to CuDEPP, DEPP has a higher theoretical capacity of 211 mAh g^−1^ (187 mAh g^−1^ for CuDEPP) based on four-electron transfer. In this study, the charge storage of sodium and its counter anions in DEPP and CuDEPP is initially studied and electrochemical performance is optimized with different content of conductive carbon. Moreover, the reaction mechanism of porphyrin-based cathode is systematically monitored using in situ XRD, ex situ XPS, IR, UV–Vis, EIS, as well as DFT simulation studies. Finally, a full cell of CuDEPP cathode and hard carbon anode is assembled to better understand the practical application properties.

## Result and Discussion

**Scheme 1:**
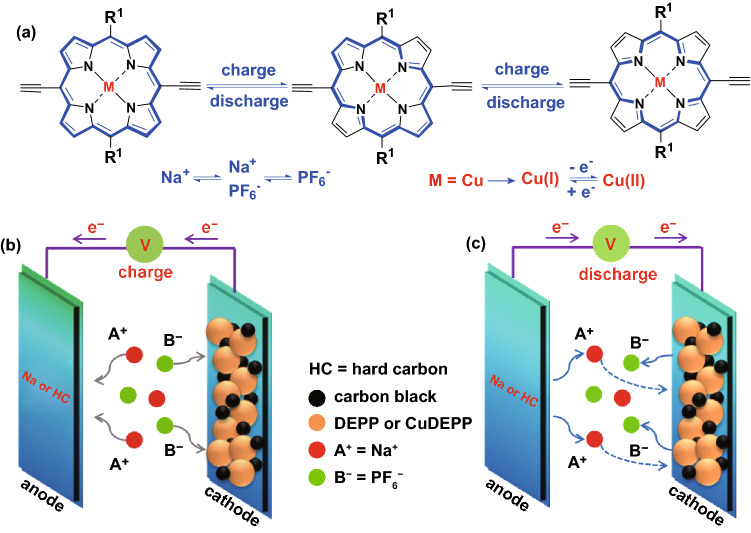
**a** Working principle of porphyrin complex during the charge and discharge process, *R*^1^ = phenyl group, *M* = 2H or Cu (denoted as DEPP or CuDEPP, respectively). **b**, **c** Schematic illustration of sodium-organic batteries with sodium metal anode and hard carbon anode, two electrons are only transferred when hard carbon is used as anode

DEPP and CuDEPP molecules were prepared via a simple synthetic route as shown in Figs. S1 and S2. The details are discussed in the experimental section in the Supporting Information. The electronic conductivity of CuDEPP and DEPP were 1.54 × 10^–4^ and 5.62 × 10^–4^ S·cm^−1^ measured by four-probe configuration, which identified the semiconductivity of the porphyrin complex. Typical IR absorption at wave numbers of 3264 and 2096 cm^−1^ were observed, assigned to –C≡C–H and –C≡C–, respectively. Cu–N bond absorption at 1070 cm^−1^ was only observed in CuDEPP and –N–H vibration of DEPP was detected at 965 cm^−1^ (Fig. S3). The Soret band of DEPP and CuDEPP at 426.6 and 426.5 nm was revealed in UV–Vis spectra, respectively. Weak peaks with wavelengths at 530.8 and 569.5 nm for DEPP and wavelengths at 561.8 and 600.6 nm for CuDEPP were observed, which are ascribed to typical Q band of porphyrin molecule (Fig. S4) [44]. SEM reveals that both DEPP and CuDEPP crystalize in rod-like morphology (Fig. S5). Nitrogen and carbon distribution in DEPP was clearly detected by EDX technique (Fig. S6). DEPP and CuDEPP showed limited solubility in common solvents such as DMC, PC; however, it showed relatively higher solubility in a highly polar molecule such as THF. After immersed in THF for two days, both DEPP and CuDEPP dissolved partially resulting in purple and green solvent (Figs. S7 and S8). Without the presence of Cu ion in the porphyrin complex, DEPP showed higher solubility in THF, indicating that the strong *π*-cation interaction of ethynyl group and Cu ion in the CuDEPP renders it good stability. Since the aromatic porphyrin molecule (18*π*) has bipolar property, it can be oxidized and reduced to antiaromatic molecules of 16*π* and 20*π*, respectively (Scheme 1a). Thus, DEPP and CuDEPP are proposed as new cathodes in sodium-organic battery and the charge storage mechanism of porphyrin-based cathode coupling with a sodium metal and/or a hard carbon anode is shown in Scheme 1b.

### Electrochemistry of MDEPP in Na-Organic Cells

**Fig. 1:**
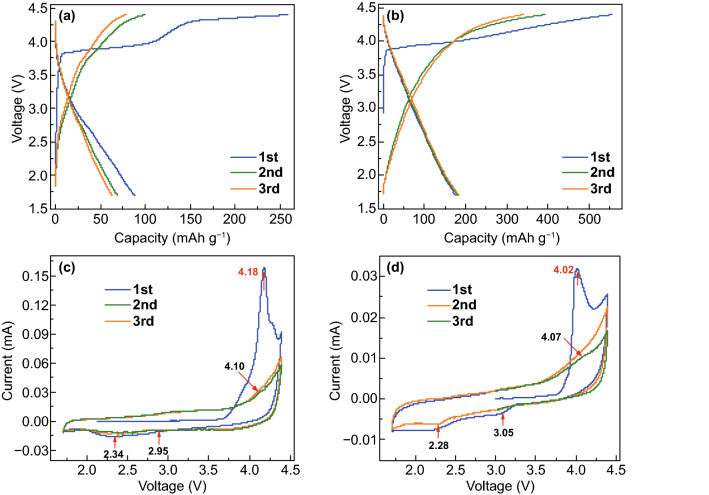
**a**, **b** Initial charge–discharge curves of DEPP and CuDEPP cathodes for sodium-organic batteries at 100 mA g^−1^. **c**, **d** CV curves of DEPP and CuDEPP cathode at a scanning rate of 0.1 mV s^−1^, the test was operated in a voltage range of 4.4–1.7 V (vs Na^+^/Na)

DEPP and CuDEPP (50 wt% of the total mass of electrode if there is no specific description) were initially tested as cathode in sodium-based half-cell, the voltage range was 4.4–1.7 V (vs. Na^+^/Na). In the first cycle, charge capacities of 258 and 550 mAh g^−1^ ( based on the mass of active material) were obtained at a current density of 200 mA g^−1^ for DEPP and CuDEPP cathodes, enabling the first discharge capacities of 85 and 185 mAh g^−1^, respectively (Fig. 1). DEPP showed two plateaus at 3.7 and 4.1 V in the first charge, while only one sloping plateau at around 4.0 V was observed for CuDEPP when sodium metal was used as anode. This process is related to the interaction of PF_6_^−^ anion with the charged DEPP and CuDEPP cathode (PF_6_^−^ doped DEPP or CuDEPP) and the self-stabilization by electropolymerization of ethynyl groups of the porphyrin molecules, which will be evidenced by spectroscopic studies and discussed later. In the second cycle, the charging curves of DEPP and CuDEPP are different from the first one, and the sloping curve was remained in the following cycles. It was observed that both DEPP and CuDEPP cathode became extremely stable after the first cycle, even in a solvent with high polarity such as THF (Figs. S9 and S10). In additional, both the purple DEPP and CuDEPP solid sample were changed to black after initial cycles. Thus, this process was of great significance in terms of the stabilization of electrodes for organic batteries. Notably, the sloping curve without discharge voltage plateau in the subsequent cycles indicated that the charge storage mechanism may be related to a fast kinetic process.

Cyclic voltammetry (CV) measurements were recorded in a voltage range of 4.4–1.7 V at a scan rate of 0.1 mV s^−1^ (vs. Na^+^/Na), where DEPP or CuDEPP were used as working electrode, sodium metal was used as counter and reference electrode. An irreversible oxidation peak was observed at 4.18 and 4.02 V for DEPP and CuDEPP, respectively, which is consistent with the charge–discharge curves. In the first cathodic scan, weak reduction peaks were observed at 2.95 and 2.34 V for DEPP, and 3.05 and 2.28 V for CuDEPP. In the following scans, oxidation and reduction curves were similar compared to the previous one, suggesting that the charge and discharge underwent the same path. Compared to the voltage gap between the oxidation and reduction reaction, relatively higher overpotential was detected in DEPP cathode. The CV test of CuDEPP cathode at a lower scan rate of 0.01 mV s^−1^ was also applied after the first charge and discharge cycle (Fig. S11). During the first anodic sweep, two oxidation peaks appeared at 3.43 and 4.06 V. In the subsequent cathodic scan, reduction peaks at 3.95, 3.48, 3.01, and 2.10 V were detected, which supported the de-interaction of PF_6_^−^ anion from CuDEPP and followed the interaction of Na^+^ in the CuDEPP. In the following anodic and cathodic cycles, oxidation peaks at 3.07 and 3.61 V and reduction peaks at 3.01 and 2.10 V were observed, corresponding to the reversible reactions [CuDEPP]^2+^  + 2e^−^ ↔ [CuDEPP] and [CuDEPP] + 2e^−^ ↔ [CuDEPP]^2−^, respectively. And the cathodic peak in 3.95 V was associated with the de-intercation of PF_6_^−^ during discharging process. It should be mentioned that the current intensity of each peak was generally weakened upon increasing the cycle numbers, implying the alteration of charge storage mechanism. The CV curve can be different by changing the scan rate, higher current density was applied, and weaker reduction peaks can only be detected.

**Fig. 2:**
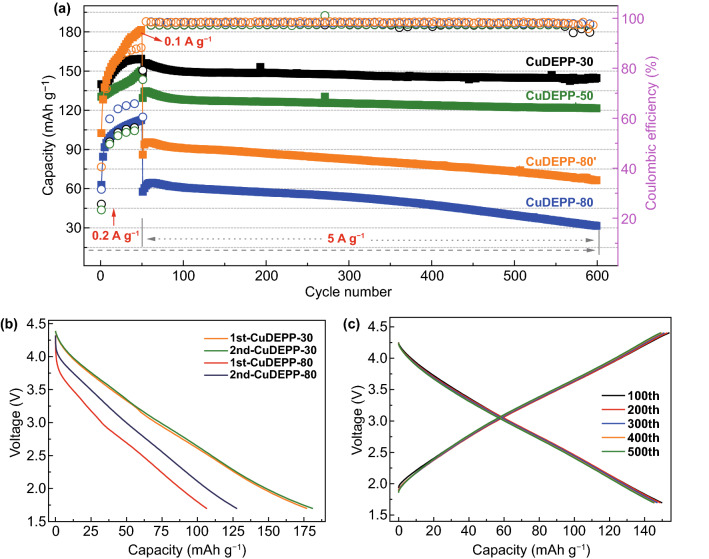
**a** Cycling performance of CuDEPP-30, CuDEPP-50, CuDEPP-80, and CuDEPP-80′ at 100 or 200 mA g^−1^ for initial 50 cycles, followed by 5 A g^−1^ for 550 cycles. **b** Discharge curves of the CuDEPP electrode of CuDEPP-30 and CuDEPP-80′ at 100 mA g^−1^. **c** Selected charge–discharge curves of CuDEPP-30 at 5 A g^−1^

Even DEPP showed high charge capacity in the first cycle, the discharge capacity was relatively low compared to that of the CuDEPP electrode. The cycling performance of DEPP cathode is shown in Fig. S12. Discharge capacity reduced from 85 (the first cycle) to 30 (the 50th cycle) mAh g^−1^ even with a higher theoretical capacity of 211 mAh g^−1^. In contrast, the CuDEPP showed much higher reversible capacity and cycling stability. This implies that copper complex ion of porphyrin molecule plays an important role for a better charge storage performance. Thus, three different electrodes with 80 wt%, 50 wt%, and 30 wt% of CuDEPP were prepared to fully demonstrate its energy storage performance and they named CuDEPP-80, CuDEPP-50, and CuDEPP-30, respectively. The cells were activated at a low current density of 200 mA g^−1^ for initial 50 cycles and then operated at 5 A g^−1^. The electrode with higher carbon black delivered higher rechargeable capacity (Fig. 2a). At a relatively low current density of 200 or 100 mA g^−1^, discharge capacities were all increased by extending the cycle number. The CuDEPP-30 showed an initial discharge capacity of 140 mAh g^−1^, following by an increase of capacity to 158 mAh g^−1^ in the 50th cycle. A reversible capacity of 147 mAh g^−1^ with a high capacity retention of 93% was observed even up to 600 cycles when the cell was cycled at 5 A g^−1^. This excellent cycling stability was also reflected by the well-coincided selected charge–discharge curves of CuDEPP-30 at 100–500th cycles which represented only tiny polarization and highly reversible capability even at a high current density during long-term cycling (Fig. 2c). The CuDEPP-50 displayed a reversible capacity of 122 mAh g^−1^ and a capacity retention of 81% up to 600 cycles. This good cycling stability and high coulombic efficiency suggested the good structural stability and reversibility of CuDEPP. The CuDEPP cycled at 100 mA g^−1^ for initial 50 cycles was denoted as CuDEPP-80′. The first discharge capacity of CuDEPP-80 was 62 mAh g^−1^, increased to 112 mAh g^−1^ in the 50th at 200 mA g^−1^. While for CuDEPP-80′, when the current density was 100 mA g^−1^, the first discharge capacity of CuDEPP-80′ was 102 mAh g^−1^ and the value was increased to 182 mAh g^−1^ in the 50th cycle, closer to the theoretical capacity based on four-electron transfer (187 mAh g^−1^). At the same current density of 5 A g^−1^, the CuDEPP delivered different reversible capacities with two different activation process. Discharge capacities of 60 and 90 mAh g^−1^ were achieved at 5 A g^−1^ in the 100th cycle for CuDEPP-80 and CuDEPP-80′, respectively. In the 600th cycles, these values were 31 and 66 mAh g^−1^, respectively. This indicates the reversible capacity of CuDEPP is largely affected by the activation process. The relatively low capacity of the CuDEPP-80 at a high current density might be caused by the insufficient content of conductive carbon for enhancing the electronic conductivity of electrode. The activation process was further monitored in the discharge curves of CuDEPP-80′ (Fig. 2b). The reversible capacity was increased from 106 to 137 mAh g^−1^ and the voltage hysteresis was significantly reduced upon cycles.

**Fig. 3:**
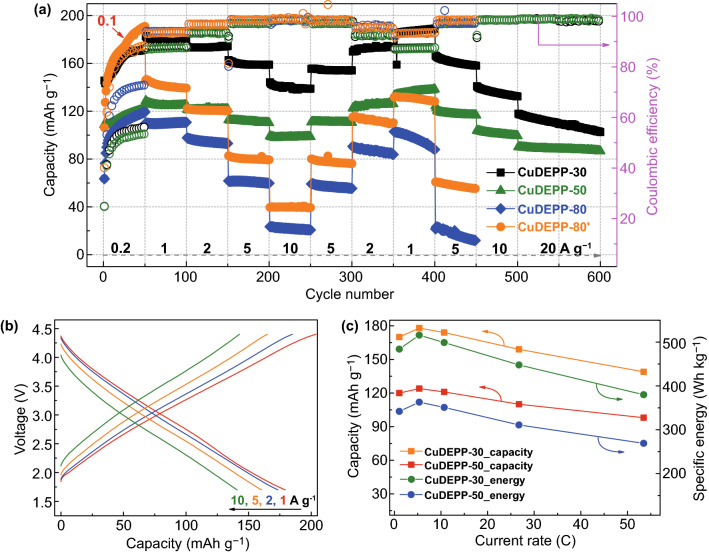
**a** Rate capability of CuDEPP electrodes with different content of active material. **b** Charge–discharge curves of CuDEPP-30 at different current densities. **c** Discharge capacity and energy density of CuDEPP-30 and CuDEPP-50 cathode at different current rates

In addition to the excellent cycling performance, the rate performance of four different electrodes were further tested from 0.2 to 10 A g^−1^ (50 cycles for each current rates, Fig. 3a). At current densities of 0.2, 1, 2, 5, and 10 A g^−1^ (corresponding to the rate of 1.07, 5.34, 10.69, 26.7, and 53.4 C), the CuDEPP-30 electrode delivered discharge capacities of 172 (50th), 178 (100th), 174(150th), 158 (200th), and 138 (250th) mAh g^−1^, respectively. The discharge capacities were recovered to its initial discharge capacity when the current density gradually returned to 1 A g^−1^, exhibiting the high reversibility of the electrochemical reaction. The excellent rate capacity with limited capacity shrinkage was also illustrated in charge–discharge curves of CuDEPP-30 at various current densities ranging from 1 to 10 A g^−1^ (Fig. 3b). Notably, as it is displayed in Fig. 3c, the specific capacity shows dependence on current rate. At the high rate of 53 C, the CuDEPP-30 electrode delivered a specific energy density of 384 Wh kg^−1^ at a specific power density of 28 kW kg^−1^ (charging in 50 s). Even at an extremely high current density of 20 A g^−1^ (106 C), it could still demonstrate a reversible discharge capacity of 109 mAh g^−1^ (550th) at an average discharge potential of 2.8 V (vs. Na^+^/Na), corresponding to a specific energy density of 288 Wh kg^−1^ and a specific power density of 56 kW kg^−1^ (charging in 18 s). As for CuDEPP-50, specific energy densities of 282 and 238 Wh kg^−1^ were delivered at C-rates of 53 and 106, respectively. Both high energy density and high power density were achieved for CuDEPP-30 and CuDEPP-50 electrodes. This can be accounted to the excellent electron transfer path and fast charge mobility at the CuDEPP surface enabling the improved electrochemical performance. While the less carbon in the electrode, high energy density can still be obtained at a low power density (Fig. 3a). However, at a high current density, both CuDEPP-80 and CuDEPP-80′ electrodes showed noteworthy capacity decay, decreased from 120 and 190 mAh g^−1^ at 0.2 A g^−1^ (the 50th cycle) to 110 and 140 mAh g^−1^ at 1.0 A g^−1^ (the 100th cycle), further decreased to 20 and 40 mAh g^−1^ at 10 A g^−1^ (the 250th cycle). This result suggested that both the ratio of conductive carbon and activation current density played a great role in exhibiting the performance of CuDEPP cathode. Higher ratio of conductive carbon in the electrode provided entirely conducting network facilitating the ionic diffusion and electronic transportation which corresponds to higher capacity and better rate capability. Therefore, through the optimization of carbon conductive and activation condition, the high rate capability and excellent cycling stability of CuDEPP electrode material can be endowed with a great prospect in sodium-organic battery.

### Reaction Mechanism of MDEPP in Organic-Na Battery

**Fig. 4:**
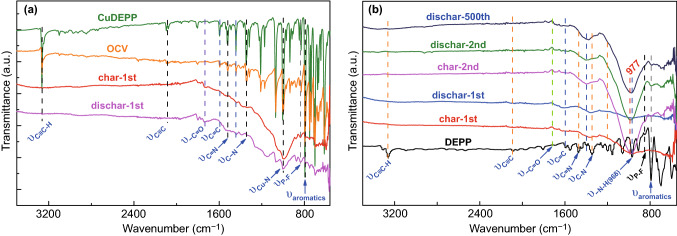
**a** Ex situ IR spectra of CuDEPP-50 and **b** DEPP cathode at different cycled states

In order to gain in-depth understanding the electrochemical performance of the CuDEPP electrode in sodium-organic ion batteries, a series of characterization methods were adopted including scanning electron microscopy (SEM), in situ X-ray diffraction (XRD), ex situ X-ray photoelectron spectroscopy (XPS), Fourier transform infrared (FTIR) spectroscopy, and UV–Vis spectroscopy. Ex situ IR study on the CuDEPP was carried out to identify the variation of porphyrin molecule during cycling (Fig. 4). The CuDEPP powder and CuDEPP electrode showed similar vibrations in a wave number range of 4000–600 cm^−1^. The stretching band at 3260 cm^−1^ and the vibration band at 2095 cm^−1^ are assigned to –C≡C–H and –C≡C– of ethynyl group of the CuDEPP. It generally disappeared in the charged state (4.4 V vs. Na^+^/Na), and it has not been recovered in the following discharged state (1.7 V). This implies clear evidence for electropolymerization of CuDEPP via the ethynyl group during charge and discharge process. The vibration peak of porphyrin at 1070 cm^−1^ assigned to Cu–N remained but broadened in the charged and discharged states compared with the as-prepared state, indicating the subtle change of electronic structure upon the π-cation interaction of ethynyl group and Cu ion in the CuDEPP molecule. The vibration band assigned to PF_6_^−^ at 838 cm^−1^ was observed in the charged state, implying the reaction of PF_6_^−^ with CuDEPP in the first charge. However, this process was partially reversible as the PF_6_^−^ vibration was still detected in the full discharged state. Absorption peak at 1580 cm^−1^ assigned to –C=N broadened in the charged and discharged states, further suggested the interaction of PF_6_^−^ anions and Na^+^ cations during cycles. It has also shown that –C=O group at 1740 cm^−1^ can also be detected in the cycled cathode which implies the formation of cathodic electrolyte interface. Without Cu complex ion in DEPP, vibration peak at 968 cm^−1^ assigned to –N–H shifted to 977 cm^−1^, and the peak broadened significantly upon cycles (Fig. 4b). Absorption bands at 1470 cm^−1^ assigned to –C=N and at 1343 cm^−1^ assigned to C–N of porphyrin merged into a broaden peak at 1398 cm^−1^. This result was strongly related to the interaction of PF_6_^−^ and Na^+^ with porphyrin molecule which bonded to nitrogen atoms of porphyrin during the electrochemical reaction. Functional groups of –C≡C–H and –C≡C– of DEPP were generally vanished upon charging and in the further discharging process, suggesting the electropolymerization of porphyrin via ethynyl group. Ex situ UV–Vis spectroscopy study was also carried out for both DEPP and CuDEPP cathode at different cycles (Fig. S13). This result revealed that Soret band of the cycled DEPP and CuDEPP showed broaden peak compared to the as-prepared sample, giving addition peaks at 434.2 and 434.9 nm, respectively. This further implies the polymerization upon charge and discharge process [45].

**Fig. 5:**
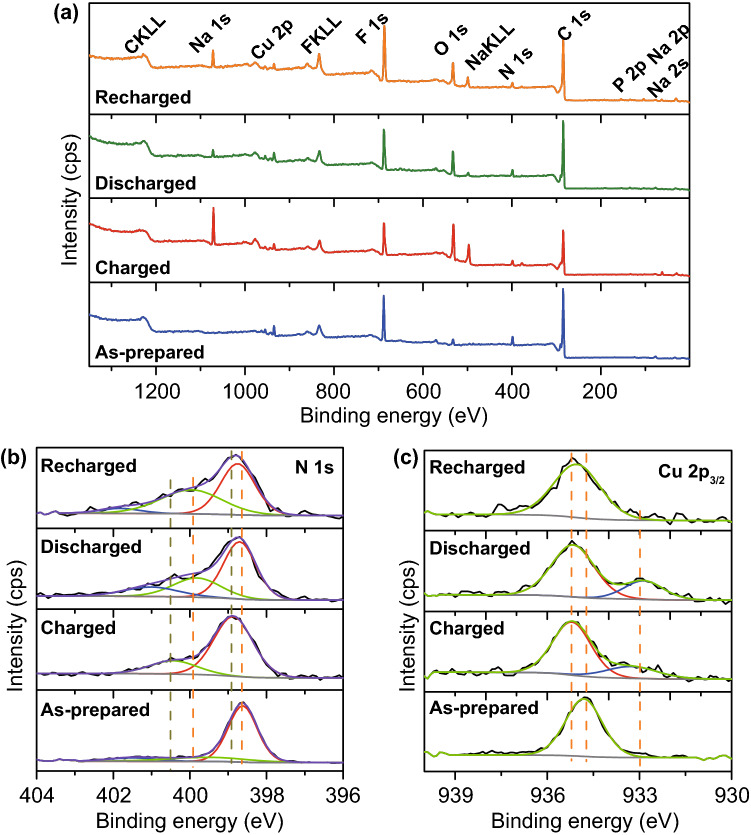
**a** Survey XP spectra of CuDEPP-50 at as-prepared, charged, discharged, and recharged states, **b** XP spectra core level of nitrogen and **c** copper of CuDEPP-50 in different cycled states

Ex situ XPS analysis was used to further investigate the oxidation state of porphyrin cathode. CuDEPP in the as-prepared, charged (4.4 V, the first cycle), discharged (1.7 V, the first cycle), and the recharged (4.4 V, the second cycle) states were carefully studied (Fig. 5). The full survey spectra of different cycled CuDEPP indicated that carbon, nitrogen, copper of porphyrin complex as well as elements of the electrolyte were detected (Fig. 5a). In the as-prepared CuDEPP, the characteristic N 1*s* with a binding energy at 398.6 eV assigned to –C=N–, while peaks with binding energy at 400.2 and 401.2 eV stand for –C–N– and the satellite feature, respectively. Upon charging, core-level spectra N1s peaks assigned to –C=N– shifted to a 398.8 eV, and it returned to 398.6 eV in the discharged states (Fig. 5b). Apart from the N 1*s* main peak of the pristine sample, additional peak at binding energy of 400.5 eV was observed for the charged sample. It shifted to 399.8 eV in the following discharged state and maintained in the recharged state. This result suggests that PF_6_^−^ and Na^+^ interacted with nitrogen atoms of porphyrin leading to the variation of N 1 s. Binding energies for Cu 2*p* at 934.7 eV (Cu 2*p*_3/2_) and 954.6 eV (Cu 2*p*_1/2_) were observed, which can be ascribed to divalent copper in CuDEPP (Figs. 5c and S14). Upon charging to 4.4 V, apart from the main peaks for Cu(II), new peak with binding energies at 932.9 eV appeared, which is attributed to the monovalent copper. This process seems to violate the basic low of electrochemical chemistry that the oxidation state of metal should not be reduced upon charging. We have repeated this data and it showed the same trend. This phenomenon is quite similar with the reductive coupling mechanism which was evidenced in anionic redox cases, where the transition metal can be triggered by anions during charge and the oxidation state of transition metal was therefore reduced [46, 47]. Xia’s group has also found the same phenomenon when PF_6_^−^ anion was adsorbed to the electrode during the charging process. It was proved that partial charge transfer from F ions to transition metal and this leads to the reduction of transition metal [48]. In the discharged state (1.7 V), peaks assigned to Cu(I) was enhanced in intensity, indicating the further reduction of Cu(II). The process was related to the de-adsorbed of PF_6_^−^ anion from 4.4 to 3.0 V and followed by adsorbing of Na^+^ cation from 3.0–1.7 V, being consistent with the slight shift of Cu 2*p* peaks to higher binding energy. In the recharged to 4.4 V, satellite feature became significant again at binding energy range of 950–940 eV and Cu(I) was almost recovered to Cu(II), indicating the reversible process of adsorption and de-adsorption of both PF_6_^−^ and Na^+^. The presence of P 2*p* and F 1*s* core-level spectra in the charged and discharged CuDEPP also supported the interaction of PF_6_^−^ and Na^+^ with CuDEPP (Fig. S14).

**Fig. 6:**
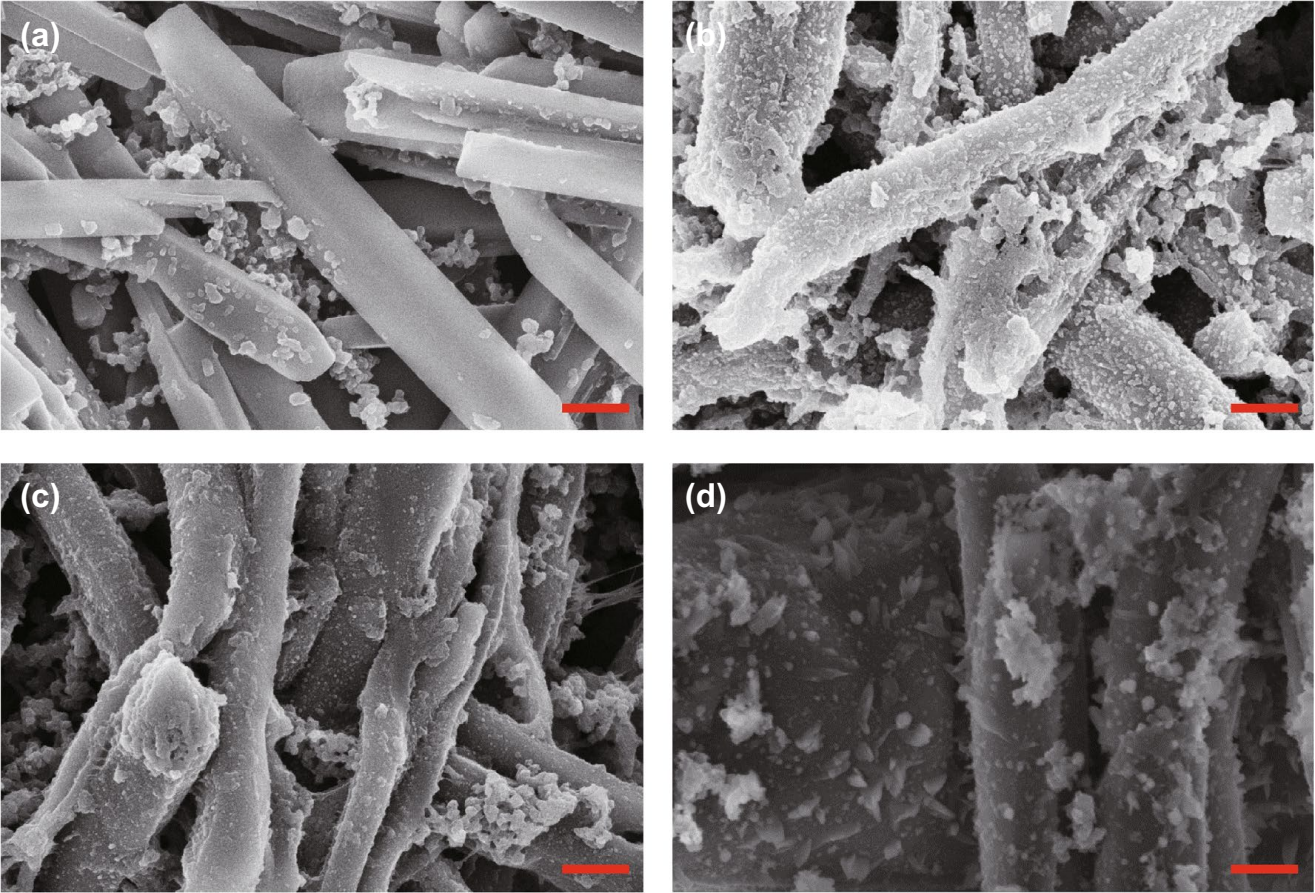
Morphology of the CuDEPP-80, **a** in the as-prepared state, **b** the first charged state, **c** in the first discharged state, and **d** in the 600th discharged state, all scale bars denote 500 nm

Morphological changes of the DEPP and CuDEPP electrode in the as-prepared, the first cycled and the 600th cycled states were characterized by SEM (Figs. 6 and S15, S16). The morphology of both DEPP and CuDEPP kept in the rod shape upon cycling; however, the rod particle has been bended after the first cycle and the surface of the DEPP and CuDEPP electrode became rough and was coated by nanoparticles (Fig. 6b, c and S15). This strongly supports the surface-involved reaction in consistent with the pseudocapacitive contribution. Moreover, the rod-like morphology maintained after 600 cycles, confirming the excellent structural stability of the CuDEPP active material (Fig. 6d). In addition, the loose crystal structure with slight pulverization and rough surfaces was observed, this was due to the expansion and shrinkage of the electrode material during the initial (dis)charging process, being consistent with the lower capacity retention of CuDEPP-80 electrode. EDX data of the charged CuDEPP showed that P and F elements were presented in the charged and discharged states. Combining with XPS and IR data, it can be concluded that the interaction of PF_6_^−^ anion with CuDEPP occurred during the charging process (Fig. S17).

**Fig. 7:**
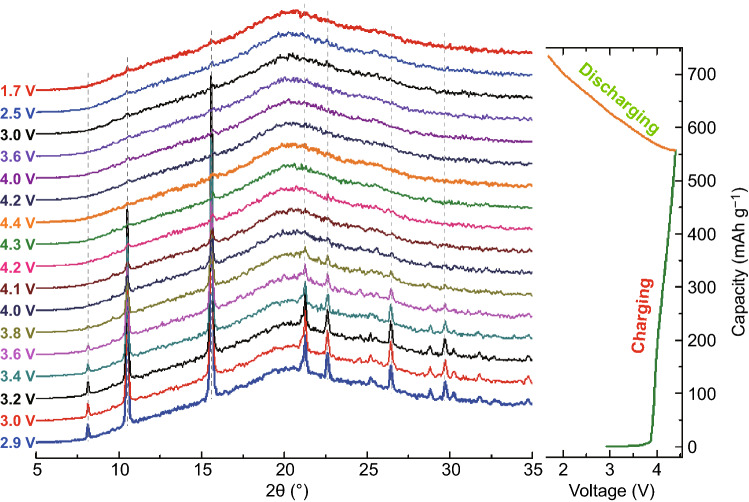
In situ XRD patterns of the CuDEPP-50 electrode during the first cycle

The CuDEPP crystalizes in triclinic system with space group ${{P}}\overline{1}$ [30], and the packing structure of CuDEPP shows that the porphyrin rings stack parallel with offset center that Cu sits between the C≡C triple bonds of the neighboring porphyrins. In order to gain more insight into the evolution of the CuDEPP crystal structure upon sodium-ion storage, in situ XRD characterization on CuDEPP electrode during the first cycle was monitored (Fig. 7). The beam of X-ray was focused at the CuDEPP cathode through the beryllium window of homemade cell. Upon the first charging process, the intensity of diffraction peaks was generally weakened and no significant diffraction patterns were detected when the cell was charged to 4.2 V (vs. Na^+^/Na). The diffraction patterns were not recovered in the following discharging process. This phenomenon implies that the interaction of PF_6_^−^ anion and followed Na^+^ cation with CuDEPP molecule broke the long-term ordered of porphyrin. The crystallinity gradually decreased during the charging process and evolved into amorphous structure without obvious peaks in the discharge state, which could be ascribed to the chemical interaction of the PF_6_^−^ and Na^+^ into the lattice of CuDEPP at the near surface [49].

**Fig. 8:**
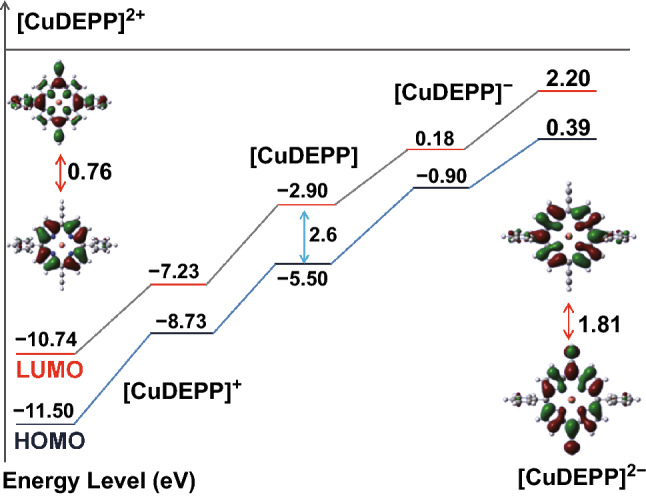
Energy levels of [CuDEPP], [CuDEPP]^+^, [CuDEPP]^2+^, [CuDEPP]^−^, and [CuDEPP]^2−^ based on DFT simulation study

Density functional theory (DFT) was used to simulate the energy of electrode in different charged states. The HOMO and LUMO values are normally given to demonstrate the feasibility of electron transfer at different redox states (Fig. 8). According to the given energy levels of [CuDEPP], [CuDEPP]^+^, [CuDEPP]^2+^, [CuDEPP]^−^, and [CuDEPP]^2−^ based on theoretical calculations, the HOMO–LUMO gap of CuDEPP was significantly reduced at both charged and discharged states compared to the as-prepared electrode. The relative low energy gap of [CuDEPP]^2+^ (0.76 eV) and [CuDEPP]^2−^ (1.81 eV) renders it highly reversible electrochemical reactions and high power density. It has been shown that the electropolymerization via the ethynyl group of CuDEPP occurred in the first charge, which enhanced the conjugative macrocycle of porphyrin. EIS results indicated that the charge transfer resistance was significantly reduced from 482.6 (before cycling) to 245.8 Ω in the first charged state and maintained upon following cycles, further supporting the improved kinetics after the initial cycle (Fig. S18). The stable structure and well-localized orbitals of CuDEPP after the first charging thus manifesting that the CuDEPP molecule can reversibly load two positive charges and two negative charges during the charge and discharge.

**Fig. 9:**
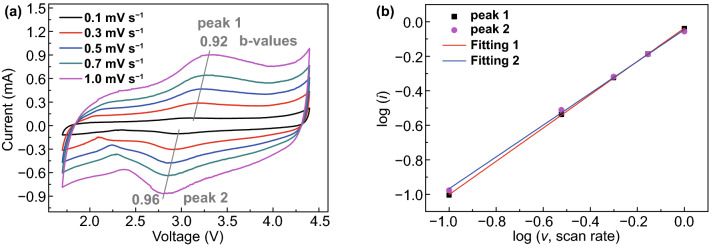
**a** CV curves of CuDEPP electrode obtained at various scan rates after initial five cycles between 1.7 and 4.4 V, **b** log (*i*, current) versus log (*v*, scan rate) for determining the b values

Extremely high power density was also achieved even large anions (PF_6_^−^) acted as the charge carriers. To explore the electrochemical kinetics of CuDEPP electrode, the cell was initially activated using galvanostatic charge and discharge for ten cycles followed by cyclic voltammetry (CV) measurements at various scan rates ranging from 0.1 to 1.0 mV s^−1^ (Fig. 9a). The shape of CV curves were non-ideal rectangular and basically retained at different scan rates. Both the cathodic and anodic peaks are broad at different scan rates after initial cycles, located at 2.8 and 3.1 V (vs. Na^+^/Na), respectively. The small gap between cathodic and anodic peak suggested high rate kinetics of CuDEPP upon electrochemical reaction. It was slightly enlarged when the scan rate was further increased, implying the increase on kinetics. To distinguish the charge contribution during the dual ion shuttle, in general, this can be quantitatively analyzed by a method proposed by Dunn and co-workers [50]. The current (*i*) obeys a power-law relationship with the sweep rate (ν), *i* = *aν*^*b*^, where *a* and *b* are adjustable parameters [51, 52]. The reaction is a capacitive-dominated charge storage if the *b* value is 1, a redox reaction controlled by semi-infinite diffusion means the *b* value is 0.5 [53]. The closer the *b* value is to 1, the greater the capacitance contribution, whereas b-value can be determined by the slope of the log (ν, scan rate)–log (*i*, current) plots (Fig. 9b). After linear fitting, the *b*-value of the anodic and cathodic peaks (denoted as peak 1 and peak 2) was determined to be 0.92 and 0.96, respectively, conforming to the kinetics of capacitive characteristics. Furthermore, the relatively low Brunauer–Emmett–Teller (BET) surface area of CuDEPP is approximately 13.01 m^2^ g^−1^, which will be helpful to suppress the occurrence of electrochemical side reactions (Fig. S19). Thus, it further indicated that charge storage of both Na^+^ and PF_6_^−^ with CuDEPP was surface-controlled reaction which enables excellent reversibility and fast charge transfer process due to the pseudocapacitive contribution.

### CuDEPP as Cathode in a Full Cell

**Fig. 10:**
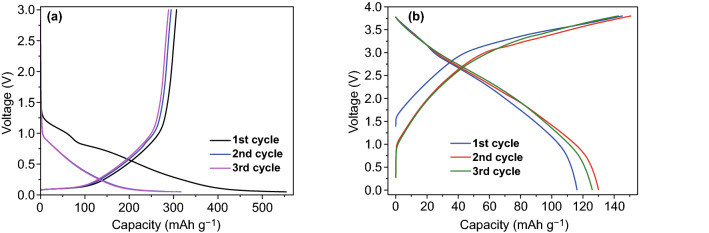
Initial charge–discharge curves of **a** Na/NaPF_6_/HC half-cell in a voltage range of 0.01–3.0 V, and **b** HC/NaPF_6_/CuDEPP full cell in a voltage range of 3.8–0.01 V at a current density of 100 mA g^−1^, where HC and the CuDEPP were used as an anode and a cathode, respectively. HC denotes hard carbon

To further test the electrochemical property of porphyrin cathode, a full cell was assembled using CuDEPP as a cathode, hard carbon as an anode (HC/NaPF_6_/CuDEPP, Scheme 1). Hard carbon was proposed as a potential anode material for sodium-ion batteries owing to its good performance and renewable raw materials. It delivered an initial discharge capacity over 550 mAh g^−1^ in Na/NaPF_6_/HC half-cell at 100 mA g^−1^ (Fig. 10a). Highly reversible sodium storage in hard carbon was observed, which renders it a good candidate as anode to match the electrochemical performance of CuDEPP. The CuDEPP cathode in conjunction with hard carbon anode was initially activated using galvanostatic charge and discharge for the electropolymerization at the cathode and the formation of solid electrolyte interphase (SEI) layer at the anode. The HC/NaPF6/CuDEPP cell delivered a first discharge capacity of 116 mAh g^−1^ and it further increased to 130 mAh g^−1^ in the third cycle (Fig. 10b,). Then, the capacity decayed in the following cycling and stabilized at 65 mAh g^−1^ in the 50th cycle (Fig. S20). The charge–discharge curves with no clear voltage plateau were obtained, which was also observed in the CV test (Fig. S20), indicating the rapid redox reaction without phase transformation. Highly reversible capability of this model full cell demonstrates that superior flexible battery with porphyrin cathode would be possible.

## Conclusions

Functionalized porphyrin DEPP and CuDEPP were proposed as new pseudocapacitive cathodes for sodium-organic battery. The study indicated that ethynyl group of porphyrin played an important role in terms of stabilization of organic electrode thereby enabling excellent cycling performance. Even DEPP has a higher theoretical capacity, it was evidenced that copper ion is needed for porphyrin cathode for better reversible charge storage performance. The reaction mechanism of porphyrin cathode was fully studied using in situ XRD, ex situ IR, UV–Vis, XPS, and DFT simulation. It revealed that the irreversibly amorphization process after the in situ electropolymerization in the first cycle caused by the interaction of PF_6_^−^ and followed Na^+^ can benefit the subsequent sodium-ion storage reaction. Pseudocapacitive domination processed by the surface-controlled reaction was partly responsible for the fast charge transfer and storage. The CuDEPP delivered an excellent cycling stability at the current density of 5 A g^−1^ (93% after 600 cycles) and high specific energy density 384 Wh kg^−1^ at a specific power density of 28 kW kg^−1^ with an average voltage of 2.8 V (vs. Na^+^/Na), based on a rapid redox conversion of four-electron transfer among CuDEPP molecule. Besides, the CuDEPP as a cathode coupling with hard carbon anode demonstrates highly reversible capability and charge storage performance. The study using small molecule of porphyrin as electrode material here can provide enormous possibilities for the development of the cost-effective and abundant organic electrode materials applied in next-generation EES. We expect that the metal porphyrins and/or phthalocyanines with ethynyl group as cathode can not only avoid the dissolution of organic active material, the electronic conductivity and the cycling stability of cathode can also be enhanced as well.

## Supplementary Information

Below is the link to the electronic supplementary material.

Supplementary material 1 (PDF 1243 kb)
