# Supplementary material for: High Rate and Long Lifespan Sodium-Organic Batteries Using Pseudocapacitive Porphyrin Complexes-Based Cathode
Source: Nanomicro Lett. 2021 Feb 14;13:71. doi: 10.1007/s40820-021-00593-8 (PMC8187698; doi:10.1007/s40820-021-00593-8)
Supplement: Supplementary file 1 — Supplementary material 1 (PDF 1243 kb) [file 40820_2021_593_MOESM1_ESM.pdf]

Supporting Information for

High Rate and Long Lifespan Sodium-Organic Batteries Using Pseudocapacitive Porphyrin Complexes Based Cathode

Xi Chen[1, #], Xin Feng[1, #], Bo Ren[1], Liangzhu Jiang[1], Hongbo Shu[1], Xiukang Yang[1], Zhi Chen[2, *], Xiujuan Sun[1], Enhui Liu[1], Ping Gao[1, *]

[1]Key Laboratory of Environmentally Friendly Chemistry and Application of Ministry of Education, Key laboratory for Green Organic Synthesis and Application of Hunan Province, College of Chemistry, Xiangtan University, Xiangtan 411105, P. R. China

[2]International Collaborative Laboratory of 2D Materials for Optoelectronics Science and Technology of Ministry of Education, Institute of Microscale Optoelectronics, College of Chemistry and Enviromental Engineering, Shenzhen University, Shenzhen 518060, P. R. China

[#] Xi Chen and Xin Feng contributed equally to this work

*Corresponding authors. E-mail: ping.gao@xtu.edu.cn (P. Gao); zhi.chen@szu.edu.cn (Z. Chen)

S1 Experimental Section

S1.1 Materials Preparation

Functionalized porphyrin complexes of 5,15-bis(ethynyl)-10,20-diphenylporphyrin (DEPP) and [5,15-bis(ethynyl)-10,20-diphenylporphinato]copper (II) (CuDEPP) were prepared according to the synthetic route as shown in Figure S1 and Figure S2. Three cathodes with 30 wt%, 50 wt%, and 80 wt% of CuDEPP active material were prepared, where the conductive carbon black were 60 wt%, 40 wt%, and 10 wt%, respectively. The polyvinylidene fluoride (PVDF, 10 wt %) was used as the binder in the cathode. The graphite and hard carbon electrodes were prepared by mixing 85 wt% active material, 5 wt% carbon black and 10 wt% PVDF binder, where stainless steel and copper foil were used as current collector respectively. The DEPP cathode was composed of 50 wt% DEPP active material, 40 wt% conductive carbon black, and 10 wt% of PVDF binder. The slurry of cathode was prepared by grinding the mixture in N-methylpyrrolidone (NMP) for 30 min, and then pasted on a 316 L stainless steel foil (16 mm in diameter). The electrodes were dried at 373 K under vacuum for 10 hours. Glass fiber filters as separator (GF/D, Whatman) were dried at 393 K under vacuum for 12 h. The mass loading of each electrode was 1.0-2.0 mg, the areal loading amount was 0.5-1.0 mg cm^{-2}. 1 M sodium hexafluorophosphate ($NaPF_6$) in propylene carbonate (PC) or in a mixture of PC and 5 wt% fluoroethylene carbonate (FEC) was used as electrolyte for the electrochemical characterization.

S1.2 Materials Characterization

1H and 13C NMR spectra were recorded with a Bruker Avance 400 instrument. UV-vis spectra of porphyrins were measured on a Perkin–Elmer Cary 60 spectrometer. Mass spectrometry (MS) data were performed on a Bruker Aupoflflex III MALDI-TOF Analyzer using CCA as matrix. The morphology and elemental analysis was observed using a field emission scanning electron microscope (FESEM, ZEISS LEO 1530) and an energy dispersive X-ray spectroscopy (EDX) with an X-Max Silicon Drift Detector. Power X-ray diffraction patterns were recorded using a D8 ADVANCE X-ray diffractometer with a Cu Ka radiation source (λ = 0.154 nm). FTIR

spectroscopy with an attenuated total reflectance model was recorded using a Thermo Fisher Nicolet IS50 spectrometer between 600 and 4000 cm^{-1}. X-ray photoelectron spectroscopy was recorded on a Escalab250Xi (Thermo Scientific), using monochromatized Al Kα radiation (1486 eV). The pass energy for survey spectra was 100 eV, for detail spectra the energy was 30 eV. The binding energies of all spectra were calibrated with respect to the C1s peak of ubiquitous carbon at a binding energy of 284.8 eV. The Brunauer−Emmett−Teller (BET) surface area was performed by an Automatic Three-station Specific Surface Area and Porosity Analyzer (TriStar II 3020). The electronic conductivity was measured by RTS-9 type 4-Point probes Resistivity Measurement System.

S1.3 Electrochemical Characterization

CR2032-type coin cells were fabricated in an Ar-filled glove box where the concentration of oxygen and water concentration was below 0.1 ppm. DEPP or CuDEPP cathodes and sodium metal anodes were separated by one piece of glass fiber in the coin cell. The charge and discharge measurements were performed with an Arbin BT2000 and an Neware battery testing system at 298 K. Cyclic voltammetry (CV) and electrochemical impedance spectroscopy (EIS) were studied using a Biologic VMP-3 and CHI660E electrochemical workstation. The voltage range for the CV measurement was 1.7-4.4 V. The frequency range for EIS was carried out between 100 KHz and 0.01 Hz with an AC oscillation amplitude potential of 10 mV.

S1.4 Computational Study

Density functional theory simulations were performed within Gaussian 09 software package using Becky's three-parameter exchange function within the Lee-Yang-Parr correlation functional (B3LYP). The geometries were thoroughly optimized for each molecule with 6-311+G(d) basis set level. Vibration spectra calculations were conducted to verify the true energy minima. The highest occupied molecular orbitals (HOMOs), single occupied molecular orbitals (SOMO) and lowest unoccupied molecular orbitals (LUMOs) were illustrated via Gauss View 3.0. A. The band gap is defined as the energy level difference between the top of the valence band and the bottom of the conduction band.

S1.5 Synthesis of DEPP and CuDEPP

S1.5.1 5,15-Bis(trimethylsilanylethynyl)-10,20-diphenyl-21H,23H-porphyrin (1)

Phenyl-2pyrrolemethane (1.332 g, 6 mmol) was dissolved in a 500 mL two-neck flask with dichloromethane (DCM) (300 mL), and then 3-trimethylsilylpropynal (0.756g, 6 mmol) was added. After degassed with dry nitrogen gas for 5 min, trifluoroacetic acid (0.2 mL, 3 mmol) was slowly dropped in and stirred. After stirred for 5 h at room temperature, the solvent was added with 2,3-dichloro-5,6-dicyano-1,4-benzoquinone (DDQ) (7 g, 30 mmol) and stirred for another 1 h. The solvent was removed under reduced pressure and the residue was purified by column chromatography (silica gel) using DCM: petroleum ether = 1:1 as eluent. The product was recrystallized from MeOH/DCM to give a purple solid (374 mg, 28%).

^{1}H NMR (500 MHz, $CDCl_3$) δ ppm 9.63 (d, J = 4.68 Hz, 4H, pyrrole-*H*), 8.85 (d, J = 4.65 Hz, 4H, pyrrole-*H*), 8.20 (d, J = 6.14 Hz, 4H, Ph-*H*), 7.94-7.73 (m, 6H, Ph-*H*), 0.63 (s, 18H, -Si(CH_3)$_3$), -2.16 (s, 2H, pyrrole -N*H*). ^{13}C NMR (126 MHz, $CDCl_3$) δppm 141.35, 134.55, 128.01, 126.94, 121.81, 106.87, 102.75, 100.88, 0.336. UV-vis (DCM, nm) 434, 508, 541, 582, 678. IR (KBr cm^{-1}) 3428, 3319, 2956, 2924(Si(C-H_3)$_3$), 2853, 2141(C≡C), 1597, 1558, 1441, 1467, 1441, 1398, 1337, 1247, 1194,

1138, 1069, 1002, 974, 964, 844, 797, 704, 657, 418. ESI-ToF Calc. for $C_{42}H_{39}N_4Si_2$: $[MH]^+$,655.3; Found: m/z 655.2.

S1.5.2 [5,15-Bis(trimethylsilylethynl)-10,20-dipheny)porphinato]copper(II) (2)

$Cu(OAc)_2·H_2O$ (520mg,2.6 mmol) was added to a solution of **1** (170 mg, 0.26 mmol) in a mixture of 100 mL THF, 100 mL DCM and 20 mL Et_3N. The reaction mixture was stirred at room temperature for 12 hours, then poured into 150 mL water and extracted by DCM (3 × 50 mL). The DCM solution was concentrated under reduced pressure and the residue was passed through a short column chromatography on silica gel (petroleum ether: DCM = 1:1). After removal of solvents in vacuum, a dark purple solid **2** (171 mg, 78%) was obtained.

UV-vis (DCM, nm) 432, 564, 606. IR (KBr, cm^{-1}) 2917(Si(C-H_3)$_3$), 2849, 2134(C≡C), 1523, 1462, 1443, 1344, 1246, 1209, 1166, 1067, 1004, 993, 840, 794, 755, 706, 666, 620, 566. ESI-ToF Calc. for $C_{42}H_{36}N_4Si_2$: $[M]^+$, 715.2; Found: m/z 715.2.

S1.5.3 5,15-Bis(ethynyl)-10,20- diphenylporphinato (DEPP)

1(143mg, 0.2 mmol) was dissolved in THF (50 mL) under an argon atmosphere at 0 °C. Then tetrabutylammonium fluoride (0.114 mg, 0.36 mmol) was added. After 30 min, the reaction mixture was poured into 50 mL MeOH. The precipitate was filtered and washed by 100 mL MeOH. The product was collected to yield a dark purple solid DEPP (84 mg, 80%). ^{1}H NMR (500 MHz, $CDCl_3$)δppm 9.63 (d, *J* = 4.68 Hz, 4H, pyrrole-*H*), 8.85 (d, *J* = 4.65 Hz, 4H, pyrrole-*H*), 8.20 (d, *J* = 6.14 Hz, 4H, Ph-*H*), 7.94-7.73 (m, 6H, Ph-*H*), 4.11 (s, 2H), -2.16 (s, 2H, pyrrole -N*H*). ^{13}C NMR (101 MHz, $CDCl_3$) δppm 141.29, 134.53, 127.98, 126.91, 121.77, 106.82, 102.70, 100.83, 77.36, 77.25, 77.04, 76.73, 0.31. UV-vis (DCM, nm) 426, 529, 570. MALDI-ToF calc. for $C_{36}H_{22}N_4$: $[M]^-$, 510.1; Found: m/z 510.0.

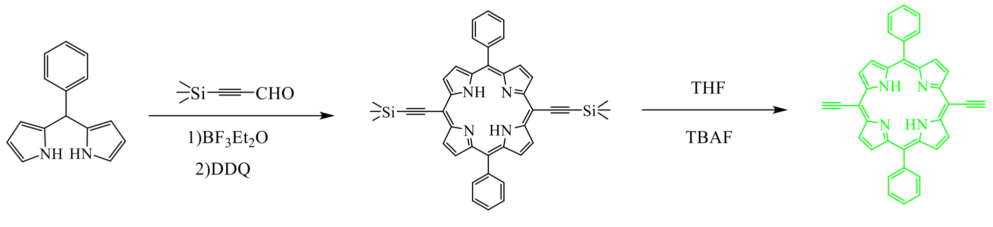

Fig. S1 Synthetic route of DEPP

S1.5.4 [5,15-Bis(ethynyl)-10,20-diphenylporphinato]copper(II) (CuDEPP)

2 (143mg, 0.2 mmol) was dissolved in THF (50 mL) under an argon atmosphere at 0 °C. Then tetrabutylammonium fluoride (0.114mg, 0.36 mmol) was added. After 30 min, the reaction mixture was poured into 50 mL MeOH. The precipitate was filtered and washed by 100 mL MeOH. The product was collected to yield a dark purple solid CuDEPP (108 mg, 95%). UV-vis (DCM, nm) 425, 558, 598. IR (KBr, cm^{-1}) 3264 (C≡C-H), 2096 (C≡C), 1596, 1521, 1443, 1347, 1211, 1174, 1070, 1004, 936, 796, 751, 737, 711, 701, 676, 666, 646, 614, 503. MALDI-ToF calc. for $C_{36}H_{20}N_4Cu$: $[M]^-$, 571.1; Found: m/z 571.0.

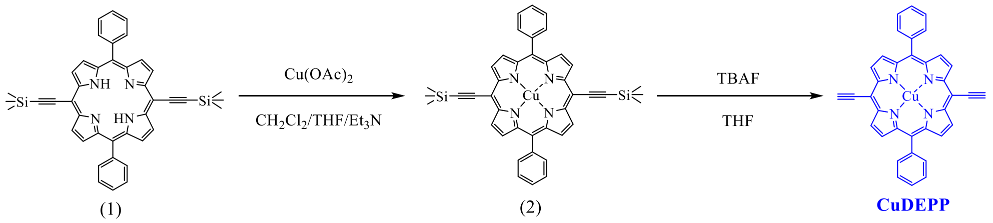

Fig. S2 Synthetic route of CuDEPP

S2 Supplementary Figures

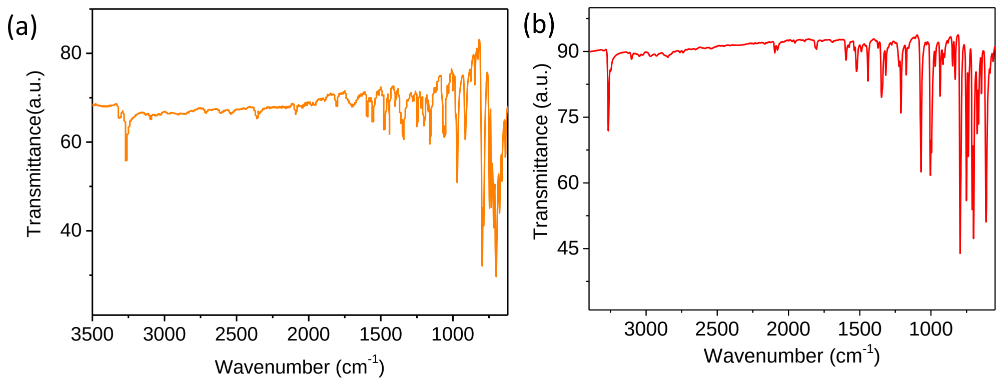

Fig. S3 IR spectra of DEPP (**a**), and CuDEPP (**b**)

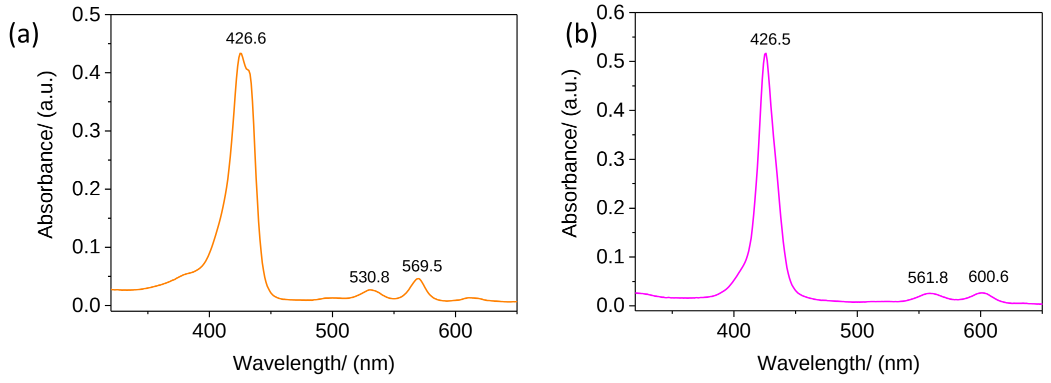

Fig. S4 UV-Vis spectra of DEPP (**a**) and CuDEPP (**b**) in THF

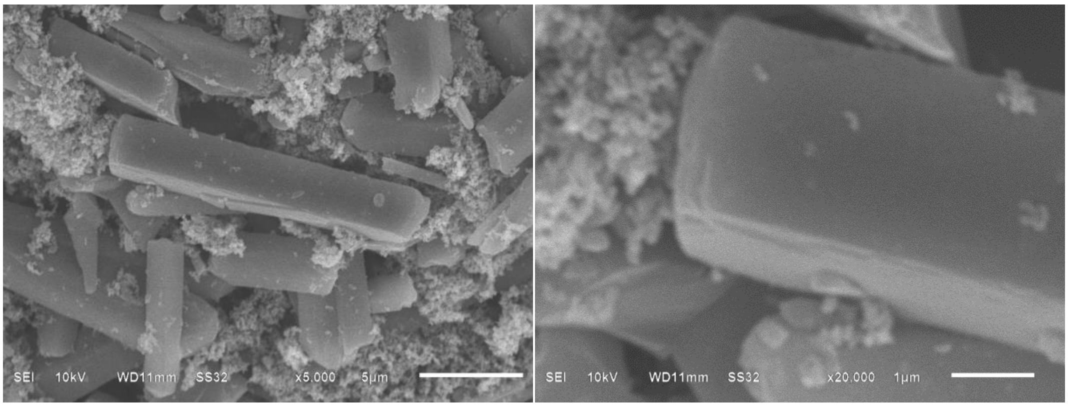

Fig. S5 SEM images of DEPP electrode

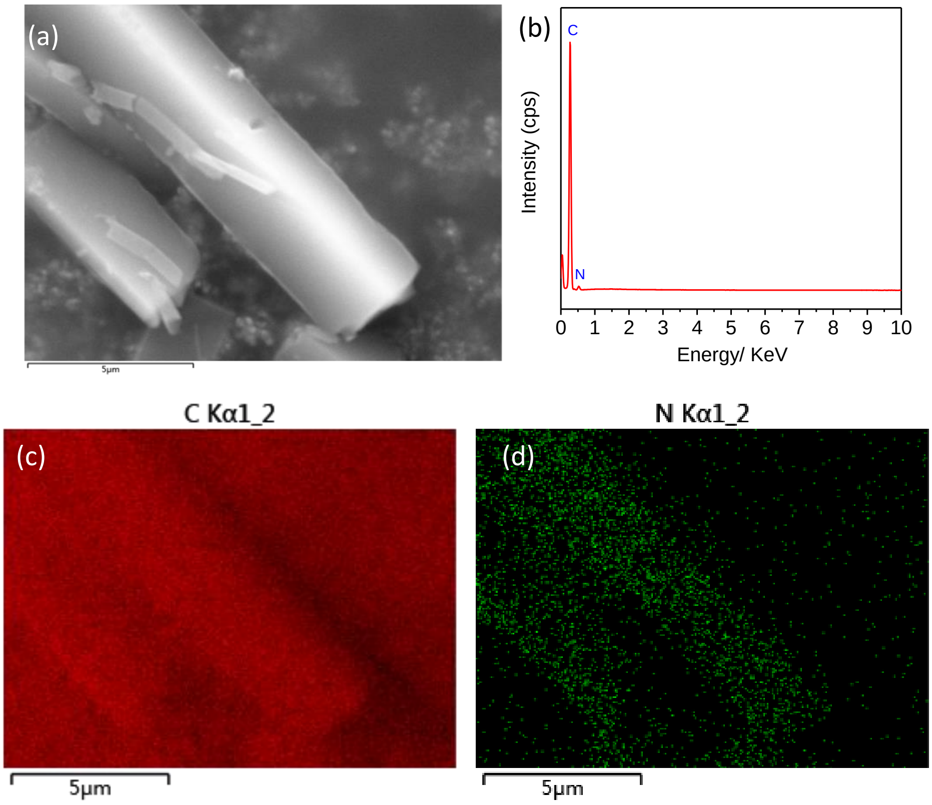

Fig. S6 Morphology of DEPP particle (**a**), EDX of DEPP (**b**), elemental mapping of DEPP (**c** and **d**)

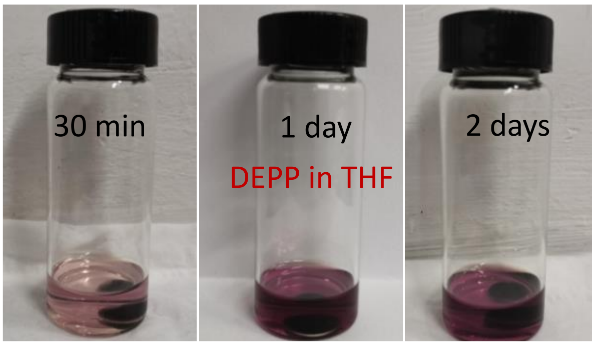

Fig. S7 DEPP electrode in THF

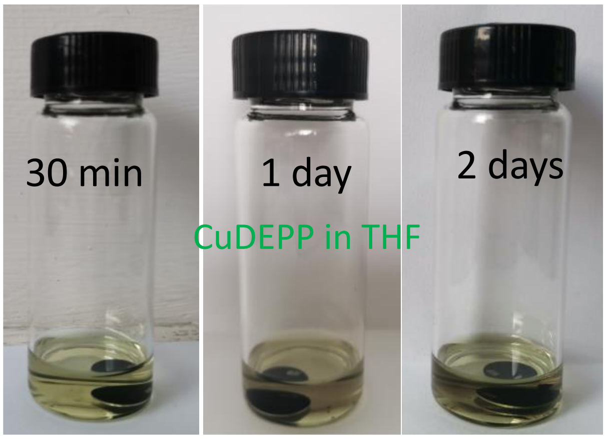

Fig. S8 CuDEPP electrode in THF

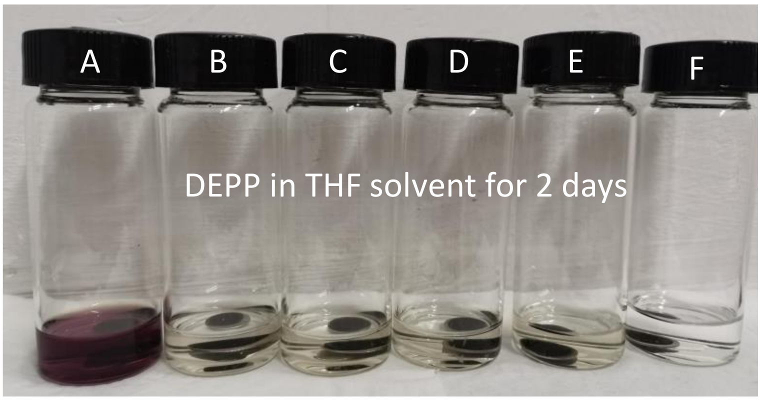

Fig. S9 DEPP in THF solvent for 2 days, **A-F** denote as-prepared, the 1st charged, 1st discharged, 2nd charged, 2nd discharged, and 500th discharged DEPP electrode

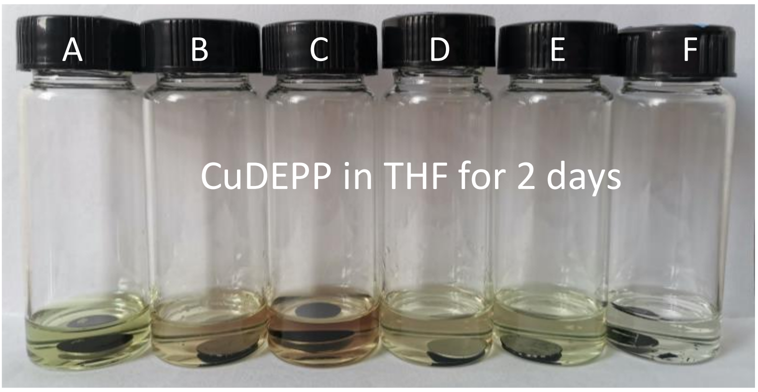

Fig. S10 CuDEPP in THF solvent for 2 days, **A-F** denote as-prepared, the1st charged, 1st discharged, 2nd charged, 2nd discharged, and 500th discharged CuDEPP electrode

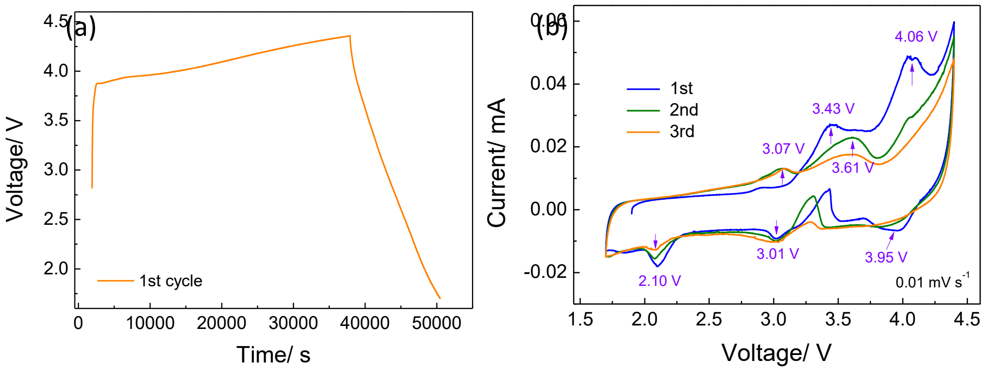

Fig. S11 (**a**) The first charge and discharge curve of CuDEPP, (**b**) CV curves of CuDEPP cathode at scan rate of 0.01mV s^{-1}

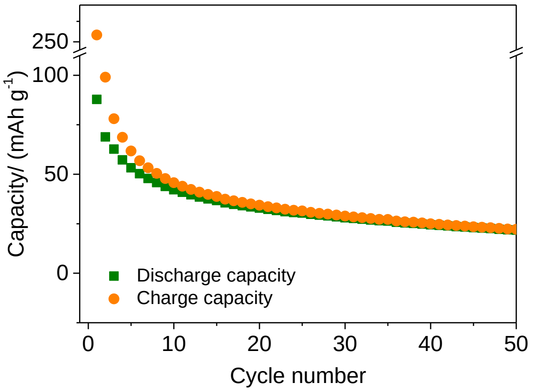

Fig. S12 Cycling performance of DEPP cathode at a current density of 100 mA g^{-1} in a voltage range of 4.4-1.7 V (vs Na^+/Na)

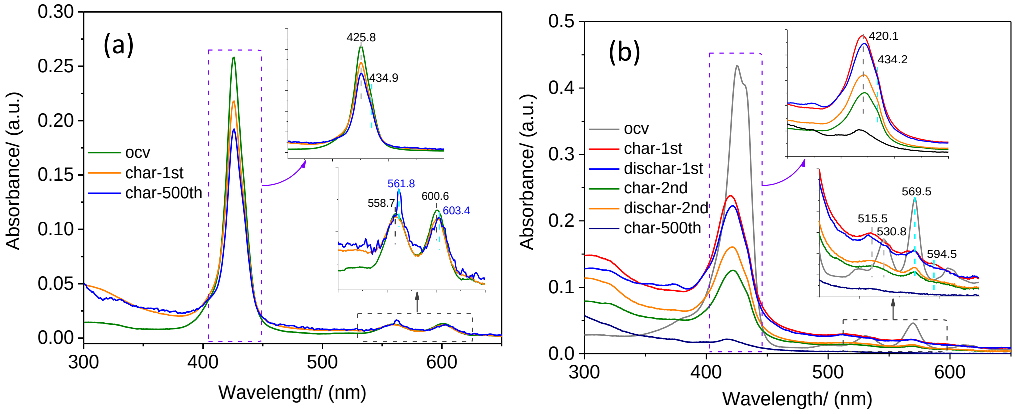

Fig. S13 UV-vis spectra of CuDEPP (**a**) and EDPP (**b**) at different states

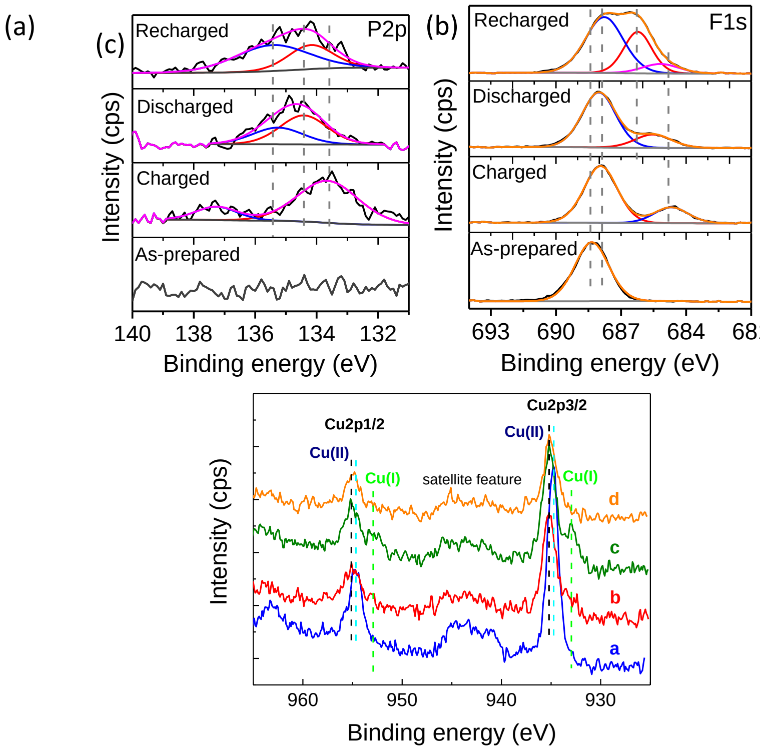

Fig. S14 XPS spectra core level of phosphorus (**a**), fluorine (**b**), and Copper (**c**) of CuDEPP in the different cycled states (**a-d** denote as-prepared, charged, discharged, and recharged CuDEPP)

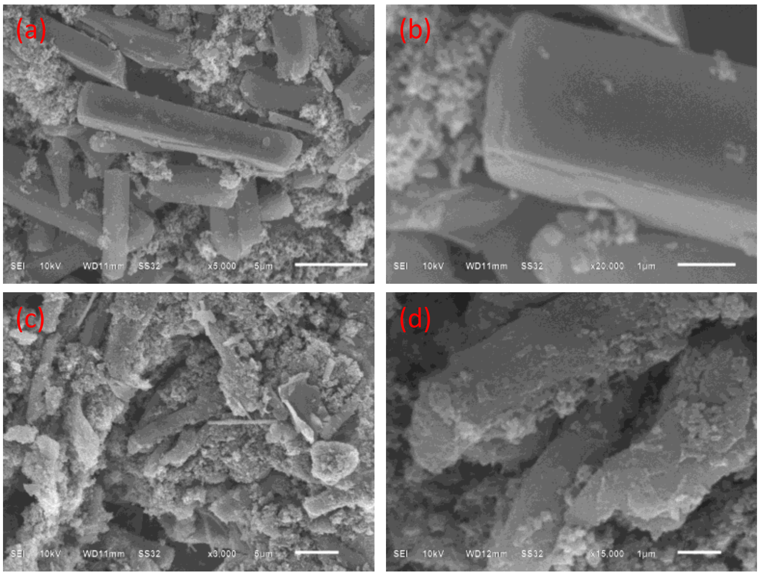

Fig. S15 Morphology of DEPP electrode, (**a, b**) in the as-prepared state, (**c, d**) in first discharged state

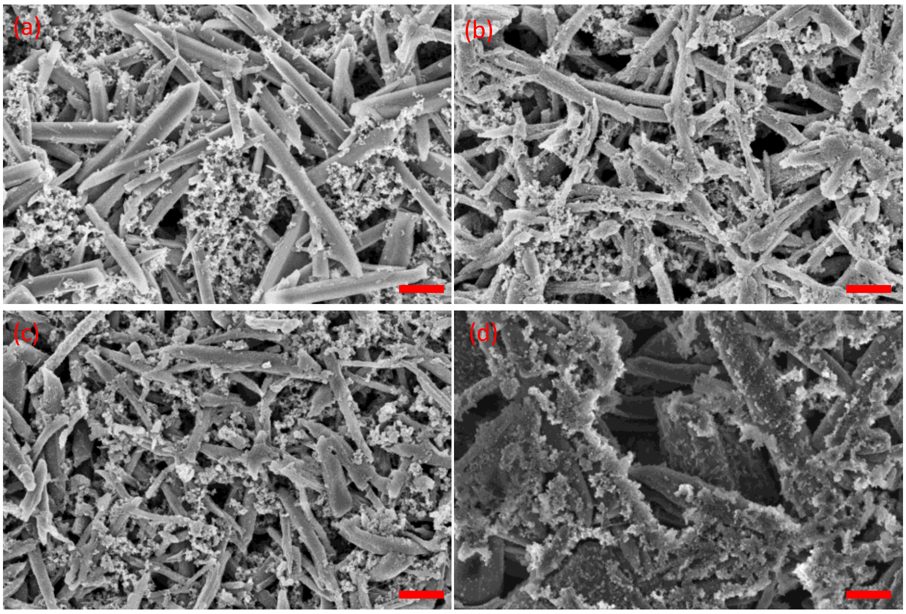

Fig. S16 Morphology of CuDEPP electrode, (**a**) in the as-prepared state, (**b**) in the first charged state, (**c**) in the first discharged state, and (**d**) in the discharged stated of the 600th cycle, the scale bar denotes 2 μm

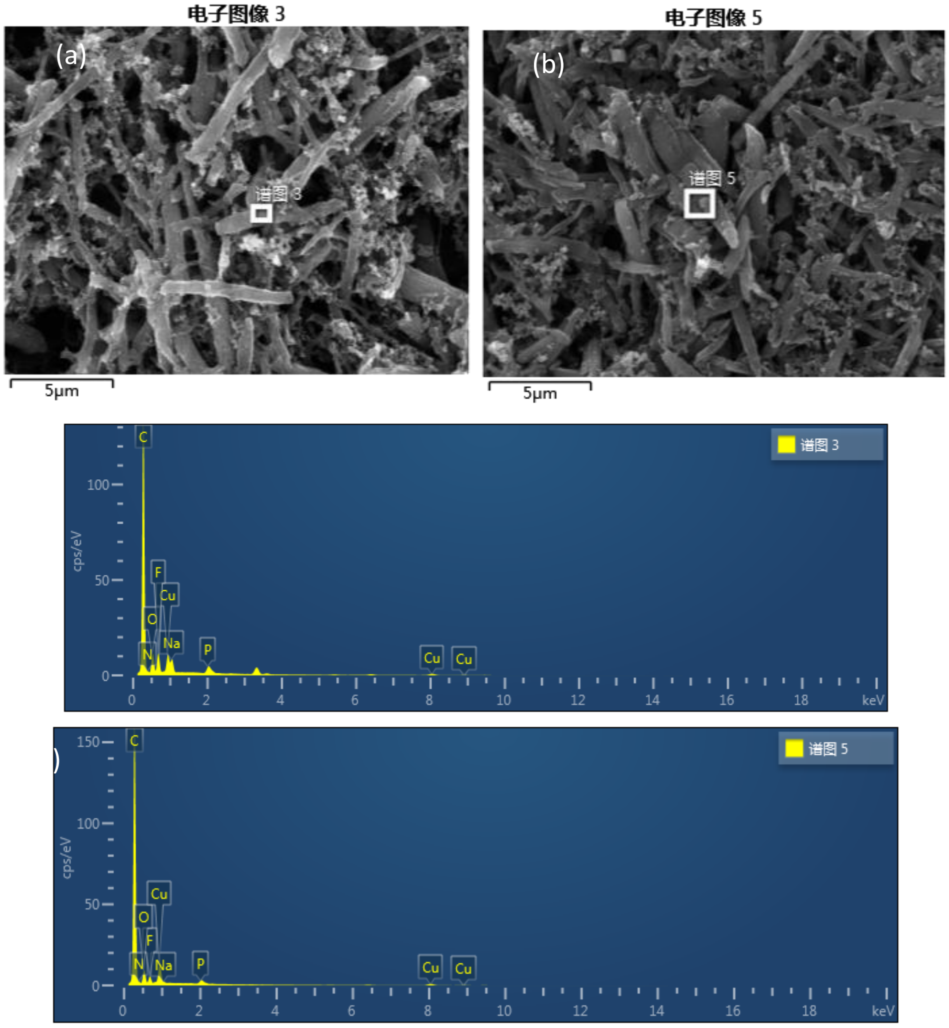

Fig. S17 SEM/EDX of CuDEPP in the 1st charged (**a, c**), and discharged (**b, d**) states

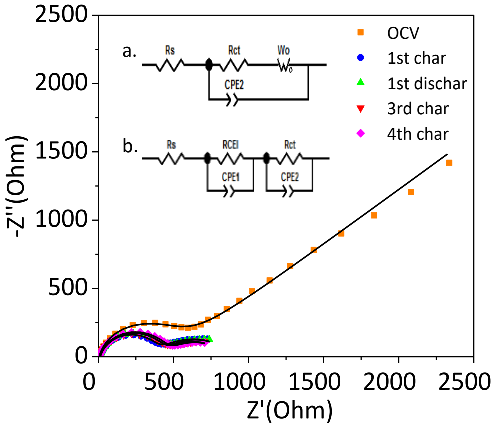

Fig. S18 EIS curves for the CuDEPP cathode at different states in initial four cycles (a, b denote equivalent circuit model for OCV and cycled sample, respectively)

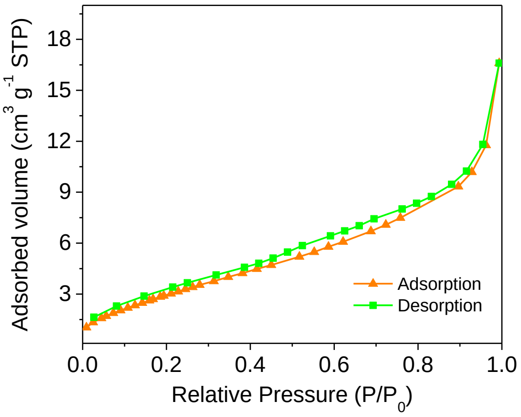

Fig. S19 N_2 adsorption and desorption curve of CuDEPP

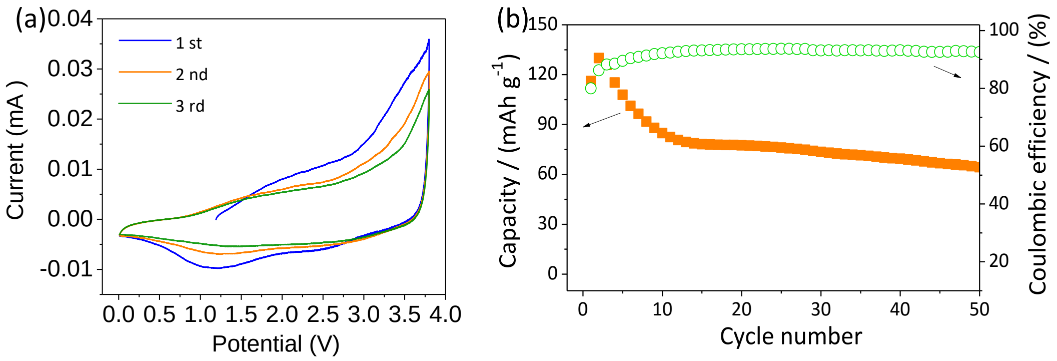

Fig. S20 CV curve (**a**) and cycling performance (**b**) of HC/NaPF6/CuDEPP full cell in a voltage range of 3.8-0.01 V at a current density of 100 mA g^{-1}
